# Highly aggressive pathology of non-functional parathyroid carcinoma

**DOI:** 10.1186/1750-1172-8-115

**Published:** 2013-08-03

**Authors:** Doina Piciu, Alexandru Irimie, George Kontogeorgos, Andra Piciu, Rares Buiga

**Affiliations:** 1 Department of Nuclear Medicine and Endocrine Tumours, Ion Chiricuţă Institute of Oncology, 34-36, Republicii St, 400015, Cluj-Napoca, România; 2 Department of Oncology, Iuliu Hatieganu University of Medicine and Pharmacy, Cluj-Napoca 400023, România; 3 Department of Pathology Athens, G. Gennimatas General Hospital, Athens, Greece; 4 Iuliu Hatieganu University of Medicine and Pharmacy, Cluj-Napoca 400023, România; 5 Department of Pathology, Ion Chiricuţă Institute of Oncology, Cluj-Napoca 400015, România

**Keywords:** Parathyroid, Cancer, Non-functional

## Abstract

Parathyroid carcinoma is a rare malignant endocrine tumor accounting for only 0.5% to 5% of all primary hyperparathyroidism. Among these malignancies, only 10-25% are nonfunctioning. After the review of the literature we could only ascertain a number of 25 cases reported worldwide, since 1929, our case being the 26th, but the first with a very aggressive pathology, treated with chemotherapy scheme usually used for neuroendocrine tumors. Considering these facts, every single case presented is a step forward in defying the clinical presentation, for the awareness of the clinicians, and also in establishing standard adjuvant therapies.

## Letters to the Editor

Parathyroid carcinoma is one of the very rare malignant endocrine tumors accounting for only 0.5% to 5% of all primary hyperparathyroidism [1]. Among these malignancies, only 10-25% are nonfunctioning [2], with normal values of parathyroid hormone (PTH). The difficulties in diagnosing these tumors rise not only because of the absence of symptoms of hyperparathyroidism, but also because of the hardship of the positive pathologic diagnosis [1-4].

After the review of literature we could only ascertain a number of 25 cases reported worldwide, since 1929 [5-31]. The present case is the 26th, being the first with a very aggressive pathology, treated with chemotherapy scheme usually used in the therapy of neuroendocrine tumors.

**Figure 1:**
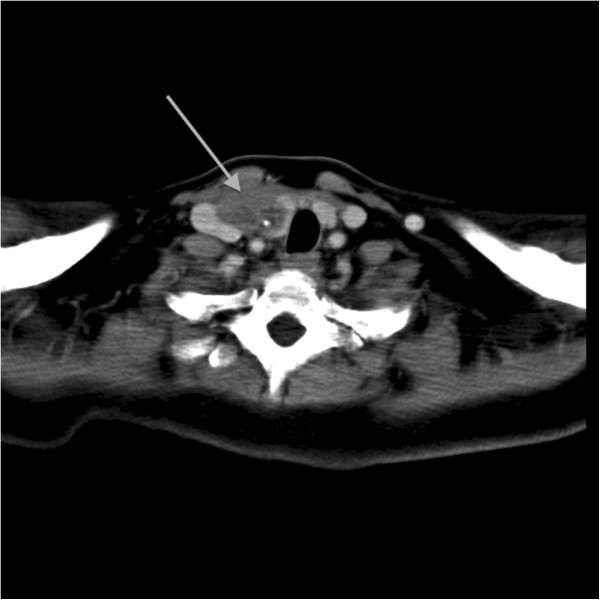
Pre-operative computed tomography of non- functional parathyroid carcinoma (arrowhead) displacing the trachea and the jugular vein, in the right anterior cervical area.

**Figure 2:**
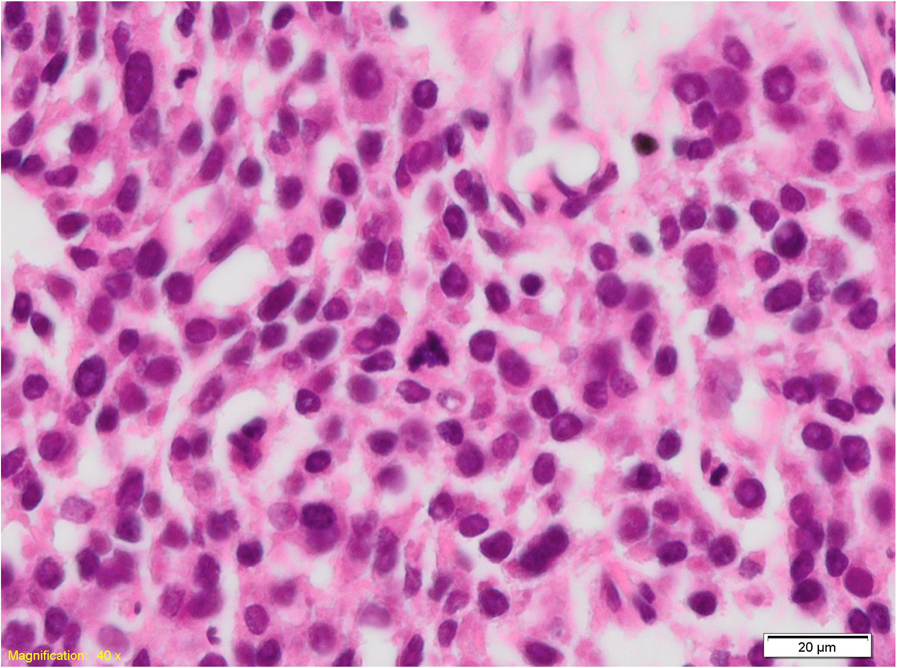
HE, 400×: Detail of nuclear atipia and multiple mitotic figures, with an atypical one in the center of the image.

We present the case of a 51-years-woman referred for an indolent cervical mass measuring 2.5 cm in the right supraclavicular area with slight evolution during the last five years. Cervical ultrasound revealed a hypoechogenic nodule located in the lower part of the right thyroid lobe, without any other lesions worth being mentioned. The serum hormones were found in normal range, as follows: thyroid stimulating hormone (TSH) was 1.21 mIU/L (N.V. 0.2-4.2 mIU/L); free-thyroxin (FT4) was 14.4 pmol/L (N.V. 12–22 pmol/L); calcium 9.1 mg/dL (N.V. 8.6-10.2 mg/dL); PTH was 54 ng/L (N.V.15-65 ng/L) and the anti-thyroid peroxidase antibodies (Anti-TPO) were negatives. Chromogranin A (Cg A) was 38 μg/L (N.V. 27–94 μg/L) and 5-hydroxiindolacetic acid (5-HIAA) was 4.2 mg/24 hours (N.V. 2–9 mg/24 hours). We performed the fine needle aspiration biopsy (FNAB) and a 2 ml volume of clear liquid was extracted, the cytology showing only colloid and amorphous material. Three years later, the patient was readmitted with a recurrence of the mass, located in the same area. The computed tomography scan (CT Scan) showed a nodule of 1 cm located in the lower part of the right thyroid lobe and another 2 cm tumor located near the thyroid gland, adherent to it and with close connection with the jugular vein (Figure 1). At this presentation, the serologic parameters were also in normal ranges. It was decided the total thyroidectomy, right selective lymphadenectomy and the radical resection of the tumor, of the jugular vein and of a part of the adjacent muscle. On Hematoxylin and Eosin (H&E) staining the tumor was composed of clear, partially oxyphil cells, showing atypia and nuclear pleomorphism with increased mitotic activity (Figure 2). At the periphery of the tumor a remnant of non-tumoral parathyroid tissue was still visible, with compression changes. The rest of the resected thyroid gland presented the microscopy of a nodular goiter, without any signs of malignancy; all the lymph nodes resected were disease free.

**Figure 3:**
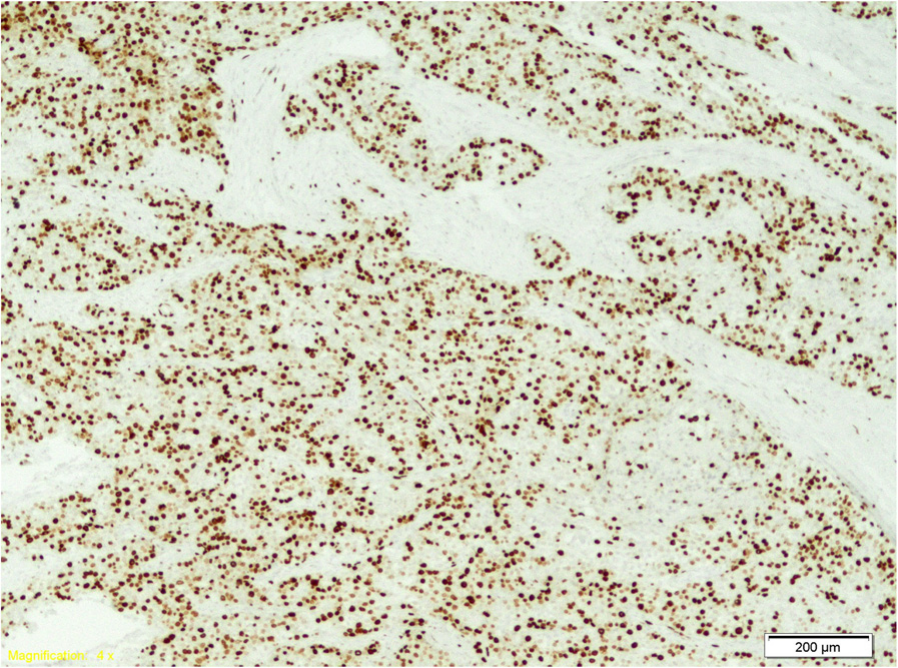
Ki67, 100×: Ki67 immunostaining, demonstrating a high proliferation index in the tumor.

The tumor cells presented the following immunohistochemical (IHC) profile: cytokeratin AE1/AE3 intensely positive; cytokeratin 7 antibody (CK7) focally positive; neuron specific enolase (NSE) intensely positive; Chromogranin A focally positive; gross cystic disease fluid protein (GCDFP15) focally positive in rare, isolated cells; progesterone receptor (PR) weakly positive (1%); estrogen receptor (ER) negative; cytokeratin 20 antibody (CK20) negative; Thyroglobulin negative; Calcitonin negative; Synaptophisin negative; Neural cell adhesion molecules (CD56) negative; Mammaglobin negative; proliferating index (Ki67) staining (Figure 3) showed a high proliferative index (70%).

**Figure 4:**
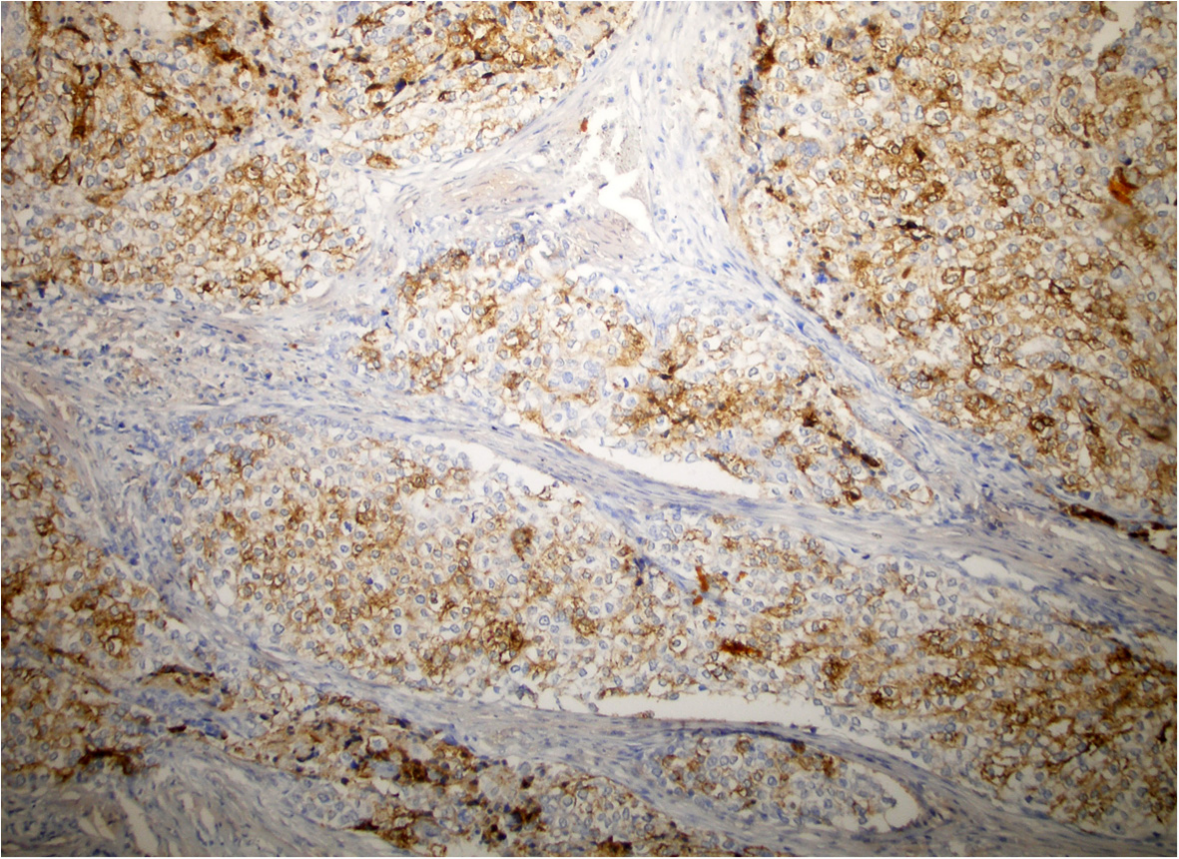
PTH, 100×: Tumor showing positive PTH immunostaining.

**Figure 5:**
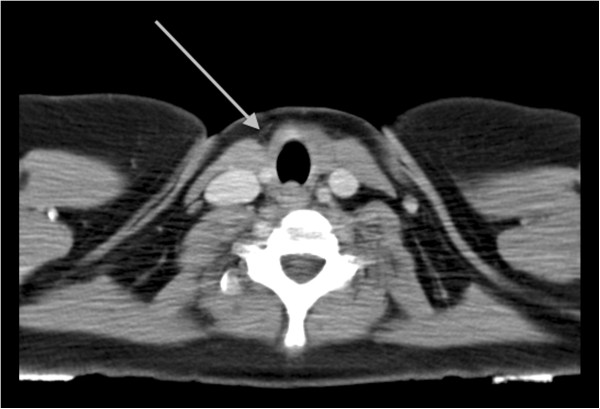
Computed tomography of the neck region, one year after therapy (surgery and chemotherapy); no local recurrence or regional metastases.

The histopathological diagnosis was probably of right parathyroid carcinoma; the normal levels of PTH and calcium enforced the PTH staining. Due to the impossibility of performing the analysis in the Institute, the accurate diagnosis was not set at that time and the block was sent for a second opinion in a national center, results showing that the PTH was negative. The confusing results misled the clinicians and made them unable to decide the treatment strategy. The capsular invasion and the infiltration of the adjacent muscle represented criteria for radiotherapy as adjuvant therapy; but the primary diagnostic of possible neuroendocrine tumor, with lack of response to external beam radiation and the presence of vascular emboli with high Ki-67 proliferation index (70%) were arguments for the chemotherapy scheme. The clinicians decided to start the chemotherapy cycles as indicated for the neuroendocrine tumors: Etoposide (Vepesid, VP16) 120 mg/sqm day 1–3 and Carboplatin AUC 5 =450 mg/day (VP16 + Carbo). A third histopathological report was demanded to the experts in Athens, Greece. Their findings indicated that PTH was extensively positive (Figure 4) and the final diagnosis was of non-functional parathyroid carcinoma. With this result, after 4 cycles of chemotherapy, the systemic treatment was discontinued. The replacement thyroid hormone therapy with 100 micrograms Levo-thyroxine daily was started and 2 months after surgery the TSH and FT4 were in normal ranges. The follow-up was carried on every 3 months and consisted in determination of the TSH and FT4, PTH, calcium and cervical CT Scan, chest CT Scan and abdominal MRI. All the examinations were constantly normal (Figure 5). The latest check-up was performed at 15 months after surgery and showed no relapse and the highly aggressive pathology did not express an aggressive clinical behavior.

In our study, the lack of reference on this pathology leaded the clinicians to establish a chemotherapy protocol (VP16+ Carbo), used for the first time in the context of this condition.

Non –functioning parathyroid carcinoma is an exceptionally rare malignancy, therefore any experience must be known, in order to improve its diagnostic and therapy.

## Competing interests

The authors declare that they have no competing interests.

## Authors’ contributions

DP has made substantial contributions to conception, acquisition, analysis and interpretation of data. AI performed the surgical procedures and participated in the design of the study. GK carried out the immunohistochemical studies and has been involved in revising the manuscript. AP elaborated the main document of the manuscript. RB carried out the pathological studies, and has given final approval of the version to be published. All authors read and approved the final manuscript.
